# Supplementary material for: Nature is resource, playground, and gift: What artificial intelligence reveals about human–Nature relationships
Source: PLoS One. 2024 Jun 17;19(6):e0297294. doi: 10.1371/journal.pone.0297294 (PMC11182501; doi:10.1371/journal.pone.0297294)
Supplement: S1 File — (DOCX) [file pone.0297294.s001.docx]

**Supplementary Materials**

*For the article:* Nature is resource, playground, and gift: What artificial intelligence reveals about human–nature relationships

Supplementary Materials Contents

[Analogies analysis 28](#_Toc120714553)

[Spectrum analysis 28](#_Toc120714554)

[Table S.1: Spectrum terms. 29](#_Toc120714555)

[Wellbeing similarity analysis 30](#_Toc120714556)

[Table S.2: Wellbeing word clouds. 30](#_Toc120714557)

[References (Supplementary Information) 31](#_Toc120714558)

# Analogies analysis additional details

One of the first and most impressive applications of word embeddings was to demonstrate their ability to detect nuanced relationships, in the form of analogies. In these famous examples, word embeddings demonstrate very high cosine similarities between, for instance, the analogy of king:queen and that of male:female; the high cosine similarities indicate that king is to queen as male is to female. Word embeddings can detect many other forms of nuanced relationship – for example, between a nation and its capital city (as distinguished from other cities located within the country)(1). We wished to see what word embeddings would reveal about collective implicit understandings of the human—nature relationship.

In order to compare analogies in a word embedding space, two pairs of words are selected. Following the method presented in (2), an analogy vector is generated by subtracting the vector of one term in a pair from the other term’s vector. The cosine similarity between the analogy vectors is then calculated, ranging from 1 (implying perfect similarity between the two analogies), through 0 (suggesting no relationship at all between the two analogies), to -1 (reflecting an opposite relationship between the two analogies; reversing the terms in one of the analogies reverses the sign on the cosine similarity measure).

We created a list of human-nature relationships based on our combined social science (RG) and humanities (AI) knowledge, and via analysis of existing typologies of human-nature relationships, as described in the introduction.

Our decision of how to run the final analogies analysis was influenced by three primary factors.

1. Many of the terms used in the literature-derived analogies were quite specific, and it was difficult to find synonyms for them. For example, it is very difficult to find appropriate one-word synonyms for “playground,” “ward,” and “warden”.
2. We ran a sensitivity analysis to determine how the analogies might shift when conducted with different permutations of the nature word-cloud used in the other two analyses. The analogies rankings shifted far more than did any of our other results (i.e., results for the other two analyses) in response to different word-cloud inputs.
3. Word cloud lists of different lengths may confuse results (see below).

For these three reasons, we decided to use individual word-pairs in our analogies analysis.

# Spectrum analysis additional details

We based this analysis on the method created by (3). That previous study created a male—female mathematical spectrum (referred to as a gender direction) using differences between word pairs (like “he-she”) in the word embedding space. It then demonstrated that this spectrum could be used to determine the implicit gendering of certain words within a language corpus. For example, the spectrum score for “lipstick” placed it near the female pole; the spectrum score for “tactical” placed it near the male pole. We wished to see how nature-related words would place on that spectrum, and on other spectra of social groupings.

We followed Bolukbasi et al.’s technique (3) of using a set of paired terms that establish the conceptual distinction we aimed to encompass; in this method, a difference vector is derived from a word pair by calculating the difference between the embedding space vectors for each of the words (e.g., “he”-“she”). Once each word pair’s difference vector was created, we then averaged these difference vectors to act as a conceptual spectrum. To measure a word’s implicit bias, we calculated that word’s cosine similarity to the spectrum. In this analysis, a cosine similarity near 1 would reflect extreme bias to one of the paired groups, near -1 would reflect bias toward the other, and 0 would reflect a complete lack of bias. Notably we chose to average the word pair vectors, rather than using the first principal component, as the principal component approach tended to favor extreme differences in single word pairs (e.g., differences in register, context, or connotation that did not reflect the specific social identities and groups intended). We also explored running analyses with every possible combination of each set of terms but found these results to be more noisy and inconsistent, similarly due to connotative nuance. For instance, the relationship between “rich” and “beggars” is quite different from the relationship between “nobility” and “beggars”, as “rich” is commonly an adjective (which is used to modify both people and a wide variety of other nouns, such as food) while “beggars” is a plural noun (with really only one meaning). In the pairings we used (Table S.SpectrumTerms), we aimed to match terms in each pair as closely as possible in context of use.

For the social groups poles of the spectra, we used the term lists in Charlesworth et al. (2022)—a study focused on how social groups appear over time in literature. We mostly used the first four words in each of their word lists (their core analysis words), but made slight modifications based on assessment of the nearest neighbor terms for each word (e.g., we excluded “asiatic” because the nearest neighbors were almost exclusively Latin binomial names for species with “asiatic” in the common name). For the ethnicity terms, we included capitalized and non-capitalized versions of each word except for “Whites”, because this was strongly associated with the All-Whites soccer team. Our final word lists are in Table S.SpectrumTerms.

Table S.1: Spectrum terms. Terms used at each pole of each spectrum for our spectrum analysis.

| **Pole 1** | **Pole 2** |
| --- | --- |
| whites | blacks |
| caucasian | negro |
| european | african |
| europeans | africans |
|  |  |
| whites | asians |
| caucasian | asian |
| european | japanese |
| europeans | chinese |
|  |  |
| whites | latinos |
| caucasian | latinas |
| european | hispanic |
| europeans | hispanics |
|  |  |
| men | women |
| man | woman |
| male | female |
| males | females |
|  |  |
| rich | poor |
| wealthy | destitute |
| aristocracy | needy |
| nobility | beggars |

# Wellbeing similarity analysis additional details

In this analysis, we compared cosine similarities between wellbeing and concepts known to be connected to wellbeing. We also included four “reference concepts” to provide context for interpretation of the cosine similarities in the core analysis (i.e., to provide a sense of baseline cosine similarities).

Sensitivity analyses suggested that word cloud size (i.e., how many words each cloud includes) might influence results: larger word clouds may tend to produce higher cosine similarities. Though this requires further exploration, to eliminate the possibility of this (potential) effect clouding our results, we used the same number of words in each concept’s word cloud.

To choose potential correlates of well-being to be included in the analysis (in addition to “nature”), we considered the wide interdisciplinary literature on human well-being, as described in the Introduction. We chose a set of concepts that consistently appear in typologies of human well-being contributors or lists of factors associated with well-being.

We choose reference concepts in two ways: first, we intentionally selected two concepts that are logically distant from well-being (electromagnetism and videogames); second, we randomly selected two concepts (pink triangles and outpost). To intentionally choose reference concepts, we brainstormed concepts that were as far as possible from both well-being and nature. Given the breadth of well-being and nature concepts, this was more difficult than it might seem at first blush. We also considered that our reference concepts needed to be encapsulated somewhat coherently by a relatively large number of words, in order to match cloud size to our other constructs. To select the random reference concepts, we used computational methods: we filtered the top 50,000 most common terms in the corpus by common nouns, then selected two using a pseudo-random number generator.

Once we had decided on target constructs, we used a six-step process to create the final word clouds (Table S.XWC):

1. We extensively discussed how to conceptualize the core concepts of well-being and nature. These are two of the most complex words in the English language, and each has diverse meanings and interpretations. We discussed the different aspects of each concept and decided on the appropriate focus for our analysis of each.
2. We pulled nearest-neighbor lists (by cosine similarity) for the words most closely connected to our target constructs (wellbeing, nature, spirituality, family, money, needs, purpose, videogames, electromagnetism)
3. When these nearest-neighbor lists made clear that the word used included meanings far from the target concept, we pulled a supplementary nearest-neighbor list to help our refinement of an appropriate word cloud. We pulled such lists for “necessities” (to accompany “needs”) and “meaning” (to accompany “purpose”).
4. Given that “nature” is central to our analysis, we conducted sensitivity analysis using different permutations of the “nature” world cloud. Results shifted in mostly subtle ways, but the general hierarchy of relationships was consistent. It was in this process that we noticed that larger word clouds seemed to result in higher cosine similarities, so we decided to standardize our word cloud sizes for the final analysis.
5. Two authors with social science and humanities training (AI and RG) assessed the wellbeing and nature word clouds jointly to determine the appropriate standardized word cloud size. They decided on 14 words and trimmed both lists to this length.
6. To build other 14-term word clouds for all constructs in the analysis, the social science and humanities authors consulted nearest-neighbor lists and refined terms until all lists had 14 terms.

Table S.2: Wellbeing word clouds. Final word lists for all constructs used in the well-being analysis.

| Construct | Words used |
| --- | --- |
| well-being | wellbeing; well-being; wellness; health; happiness; vitality; healthiness; health-related; healthful; healthy; healthier; contentment; quality-of-life; prosperity |
| nature | nature; natural; environment; environmental; landscape; wildlife; wilderness; animals nonhuman; non-human; ecology; ecological; flora; fauna |
| spirituality | spirituality; spiritual; religion; faith; religious; religiosity; holiness; sacred; spiritually; divinity; divine; sacredness; salvation; prayer |
| basic needs | shelter; food; necessities; basic_necessities; bare_necessities; basics; food_stuffs; requirements; nourishment; sustenance; subsistence; foodstuffs; basic_needs; bare_essentials; |
| purpose | purpose; goals; conviction; meaning; motivation; motivations; aim; aims; mission; purposes; intention; raison_d'_etre ; rationale ; aspirations |
| social relationships | family; friends; friend; neighbours; neighbors; community; friendship; kin; familial; neighborhood; buddies; relatives; companions; friendships |
| money | money; cash; dollars; income; wealth; financial; monetary; wealthy; dollar; fortune; funds; investments; profits; savings |
| electromagnetism (strategically selected) | electromagnetism; electromagnetic; magnetism; magnetic_fields; magnetic; electrons; electron; photons; ions; protons; electron_spin; magnet; electromagnet; superconductor |
| videogames (strategically selected) | videogame; Wii; Nintendo; Xbox; Grand_Theft_Auto; gaming; PS3; Playstation; Mortal_Kombat; Videogames; videogaming; gamers; videogamers; videogame_consoles |
| pink triangles (randomly generated) | pink triangles; yarmulkes; tzitzit; kippas; kippahs; rainbow_sashes; fezzes; Guy_Fawkes_masks; kipa; kaffiyehs; knitted_skullcaps; kipah; wear_yarmulkes; kippah |
| outpost (randomly generated) | outposts; enclave; garrison; beachhead; Nevatim_air; frontier; watchtower; hamlet; Baghran_Valley; Mitzpe_Yitzhar; Uzbin_Valley; Priestess_Maggie_Q; encampment; outpost |

# References (Supplementary Information)

1. T. Mikolov, W. Yih, G. Zweig, Linguistic regularities in continuous space word representations in *Proceedings of the 2013 Conference of the North American Chapter of the Association for Computational Linguistics: Human Language Technologies*, (2013), pp. 746–751.

2. T. Mikolov, I. Sutskever, K. Chen, G. S. Corrado, J. Dean, Distributed representations of words and phrases and their compositionality. *Advances in neural information processing systems* **26** (2013).

3. T. Bolukbasi, K.-W. Chang, J. Y. Zou, V. Saligrama, A. T. Kalai, Man is to Computer Programmer as Woman is to Homemaker? Debiasing Word Embeddings in *Advances in Neural Information Processing Systems*, (Curran Associates, Inc., 2016).
