# Supplementary material for: Nature is resource, playground, and gift: What artificial intelligence reveals about human–Nature relationships
Source: PLoS One. 2024 Jun 17;19(6):e0297294. doi: 10.1371/journal.pone.0297294 (PMC11182501; doi:10.1371/journal.pone.0297294)
Supplement: Attachment — Submitted filename: R2R PLoS.docx [file pone.0297294.s003.docx]

**Response to Reviewers**

We respond to the editorial comments in plain text (because they are quite lengthy and would not work well in a table. We then respond to the reviewer comments in the table below.

**Editorial Comments and Responses**

When submitting your revision, we need you to address these additional requirements.

1. Please ensure that your manuscript meets PLOS ONE's style requirements, including those for file naming. The PLOS ONE style templates can be found at

<https://journals.plos.org/plosone/s/file?id=wjVg/PLOSOne_formatting_sample_main_body.pdf> and

<https://journals.plos.org/plosone/s/file?id=ba62/PLOSOne_formatting_sample_title_authors_affiliations.pdf>

We have ensured that our manuscript meets these guidelines.

2. In your Methods section, please include additional information about your dataset and ensure that you have included a statement specifying whether the collection and analysis method complied with the terms and conditions for the source of the data.

We have included additional information about our dataset.

3. Please note that PLOS ONE has specific guidelines on code sharing for submissions in which author-generated code underpins the findings in the manuscript. In these cases, all author-generated code must be made available without restrictions upon publication of the work. Please review our guidelines at <https://journals.plos.org/plosone/s/materials-and-software-sharing#loc-sharing-code> and ensure that your code is shared in a way that follows best practice and facilitates reproducibility and reuse.

We have specified that our data are publicly available.

4. Thank you for stating the following in the Acknowledgments Section of your manuscript:

“We are grateful to the Gund Institute for the Environment, which provided funding for this project.”

We note that you have provided funding information that is currently declared in your Funding Statement. However, funding information should not appear in the Acknowledgments section or other areas of your manuscript. We will only publish funding information present in the Funding Statement section of the online submission form.

Please remove any funding-related text from the manuscript and let us know how you would like to update your Funding Statement. Currently, your Funding Statement reads as follows:

“This study was funded by the Gund Institute at the University of Vermont (<https://www.uvm.edu/gund>). Authors RG, NC, and AI received the funding. The funder played no role in study design, data collection or analysis, decision to publish, or manuscript preparation.”

Please include your amended statements within your cover letter; we will change the online submission form on your behalf.

We have removed funding details from the Acknowledgements, and our Funding Statement is correct.

5. We note that you have indicated that data from this study are available upon request. PLOS only allows data to be available upon request if there are legal or ethical restrictions on sharing data publicly. For more information on unacceptable data access restrictions, please see <http://journals.plos.org/plosone/s/data-availability#loc-unacceptable-data-access-restrictions>.

In your revised cover letter, please address the following prompts:

a) If there are ethical or legal restrictions on sharing a de-identified data set, please explain them in detail (e.g., data contain potentially sensitive information, data are owned by a third-party organization, etc.) and who has imposed them (e.g., an ethics committee). Please also provide contact information for a data access committee, ethics committee, or other institutional body to which data requests may be sent.

b) If there are no restrictions, please upload the minimal anonymized data set necessary to replicate your study findings as either Supporting Information files or to a stable, public repository and provide us with the relevant URLs, DOIs, or accession numbers. For a list of acceptable repositories, please see <http://journals.plos.org/plosone/s/data-availability#loc-recommended-repositories>.

We will update your Data Availability statement on your behalf to reflect the information you provide.

We have addressed these prompts.

6. Please review your reference list to ensure that it is complete and correct. If you have cited papers that have been retracted, please include the rationale for doing so in the manuscript text, or remove these references and replace them with relevant current references. Any changes to the reference list should be mentioned in the rebuttal letter that accompanies your revised manuscript. If you need to cite a retracted article, indicate the article’s retracted status in the References list and also include a citation and full reference for the retraction notice.

We have no retracted papers in our reference list.

**Reviewer Comments and Reponses**

| Reviewer #1: |  |
| --- | --- |
| From my view, the authors' methods are sound and their analyses effective. The writing is generally intelligible with few typos or errors. The general arguments and novel approaches have potential for great contribution to the subject areas in question. | Thank you. |
| I am, however, recommending minor (possibly major) revisions to the structure of the manuscript. The main reason for this is that I believe the team is trying to accomplish too much in just one paper, given its current framing. In my understanding, the authors are attempting to 1)establish the robustness of human-environment relationships literature 2) provide a novel approach to analyzing that robustness through the use of NLP methods, particularly word embedding, and 3) introduce novel approaches to NLP borne of their own interdisciplinary model. Both subject areas they are contributing to (Human-Envi. Relationships and NLP) are quite deep, and I think the their stated goals for the paper in either space are not fully met. They either lack specificity or relative robustness in the results as explained, and thus need further expounding to convince me otherwise, which will be difficult to do in just one paper. This undertaking might deserve multiple papers in its current framing. I suggest the authors either reduce scope or reframe more deeply onto one or two aspects (e.g. just the demonstration of the NLP methods' effectiveness or just the robustness of the literature that produces their analogies and how those are better grounded through NLP). See attached pdf with more comments. In short, I think the work itself is sound and should be eventually published, just with more comprehensive argumentation/presentation. | See our response below, to the closely connected, but slightly more specific, comment this reviewer left in the manuscript. |
| ***[Below are comments from R1’s PDF document, and responses]*** |  |
| R1 offered numerous helpful editorial comments that involved minor revisions (e.g., slightly clarifications of language) | We made approximately five minor revisions in direct response to reviewer suggestions in the PDF (we do not detail these below). |
| Make explicit which “environmental issues” in L47 | We added a parenthetical comment that lists specific issues. |
| Better define “implicit collective understandings” | We added a definition |
| Include references related to perspetivism – e.g., Vivieros de Castro | We have added “classic” Vivieros de Castro reference |
| Not convinced that scholarship on wellbeing has increased in the last few decades | We removed the claim that scholarship on wellbeing has increased recently; we would have to explain the “vein” of wellbeing research we intended here, and that is a red herring in this manuscript. |
| What does it mean to demonstrate something “with great complexity”? | We have changed this sentence to read “demonstrate with great nuance” |
| When are you defining the advent of the internet to be? | We do not see it as necessary to define this for this statement to make sense. The point is that the corpora include anything on the internet; when we define its beginnings is irrelevant. |
| The discussion and conclusion sections here, while well-supported by your findings, bring to attention what I think is the most significant problem which needs to be addressed: the conflict between what is professed novelty and replication. The authors claim in the outset to be presenting novel methods that produce novel interpretations, yet the results seem to suggest a supposed alignment with an already well established literature, the interactions with which doesn't seem to be given enough attention. At the same time, the literature review that conceptualizes well established concepts in human-environment relationships seems to in actuality be presented in a somewhat novel framing, one which suggests the justification for the analogies used but never really makes that justification explicit (e.g. why those analogies? where exactly in the literature do those come from? those are not clearly known)  At the same time, the introduction of novel applications or approaches to NLP methods (analogies, semantic spectra, etc.) are only lightly touched on, and their broader application only briefly suggested. I believe this subject alone could merit an entire paper in and of itself, as the authors are offering a very useful and potentially insightful approach without really giving the method or outcomes the space they deserve. I suggest the authors rethink the structure and/or scoping of the content. Perhaps it could be broken into two papers that give proper expansion to each of the problems I've posed. Or not, it could certainly still work as one paper if the scope were made less ambitious. | We appreciate this comment, as it addresses what is indeed a fundamental conundrum with this work (i.e., how to treat alignment between results acquired through these new NLP formats and results acquired through more “traditional” means). We have worked hard in our revisions to address what this reviewer perceives as a paper that attempts to do too much. Our primary response is that we did not intend for our review of well-established concepts in human-nature relationships to be presented as a novel framing. Our intention for the paper was the reviewer’s second point – the introduction of novel applications or approaches of NLP. In our revisions, we have attempted to make this clear. |
| There is a now fairly robust literature in digital humanities that utilizes NLP/ML for textual analysis, so this view [of a “virtuoso human interpreter”] is a little dated. Would add more citations of that literature to this commentary | We have added a citation to the 2022 Handbook of the Digital Humanities, which treats machine learning in some depth. |
| Define english-speaking culture here. Wolloch's work ostensibly covers not just english speakers but most of Europe over time. And also do we really mean Anglo here? | We have changed this sentence to refer to mainstream Anglophone literature, and we have added a citation to Worster 1994 to clarify. |
| Corroborate this claim of nature as gift-giver being more conducive to sutainability. This is not self evident. Needs citation. Or is it Pascual and Diaz that corroborate this? | It is indeed Pascual and Diaz (citations) that corroborate this point. We have made this more clear in our revisions. |
| A call of this stature should probably include a fairly thick description of what future action might look like. What exactly is meant by creating new repositories? Who will do it? and how? Define diversity? etc. | We have added detail to our call for a collective effort to collect and curate corpora. |
|  |  |
| Reviewer #2: |  |
| I've had the opportunity to review your manuscript on the patterns of human-nature relationships as interpreted from an extensive internet dataset. Firstly, I'd like to commend you on the clear structure of your paper; it greatly facilitates understanding and allows the reader to follow your arguments with ease. | Thank you. |
| Your novel use of NLP techniques in the realm of social science is particularly commendable. The three specific techniques you've applied have not only provided fresh insights into the topic but have also showcased the promising potential of integrating advanced AI methodologies into traditionally qualitative fields. This interdisciplinary approach is both innovative and inspiring. | Thank you, again. |
| While the methodology is detailed, a minor suggestion would be to consider incorporating a workflow diagram or flowchart. Such a visual representation would offer a clearer, concise overview of your AI-assisted approach, aiding readers in comprehending the technical processes involved more readily. | We have added this diagram (Figure 1). |
| In conclusion, your work is a valuable contribution to both social science and the growing field of AI research. I look forward to its further refinement and eventual publication. | We are very grateful for this positive review with the excellent idea of a process diagram. |
